# Supplementary material for: Modeling glioblastoma heterogeneity as a dynamic network of cell states
Source: Mol Syst Biol. 2021 Sep 16;17(9):e10105. doi: 10.15252/msb.202010105 (PMC8444284; doi:10.15252/msb.202010105)
Supplement: Supplementary file 6 — Source Data for Figure 5 [file MSB-17-e10105-s004.zip › Figure5A_sourcedata/GSEA_3071/hallmarks_stateII.GseaPreranked.1623742536692/HALLMARK_INFLAMMATORY_RESPONSE.html]

Details for gene set HALLMARK\_INFLAMMATORY\_RESPONSE[GSEA]

|  || Dataset | state23071 |
| Phenotype | NoPhenotypeAvailable |
| Upregulated in class | na\_pos |
| GeneSet | HALLMARK\_INFLAMMATORY\_RESPONSE |
| Enrichment Score (ES) | 0.27460334 |
| Normalized Enrichment Score (NES) | 1.4044449 |
| Nominal p-value | 0.05596107 |
| FDR q-value | 0.12095666 |
| FWER p-Value | 0.761 |
Table: GSEA Results Summary

  

Fig 1: Enrichment plot: HALLMARK\_INFLAMMATORY\_RESPONSE      
 Profile of the Running ES Score & Positions of GeneSet Members on the Rank Ordered List

  

| PROBE | GENE SYMBOL | GENE\_TITLE | RANK IN GENE LIST | RANK METRIC SCORE | RUNNING ES | CORE ENRICHMENT || 1 | EDN1 |  |  | 6 | 1.670 | 0.0502 | Yes |
| 2 | F3 |  |  | 13 | 1.555 | 0.0969 | Yes |
| 3 | LIF |  |  | 64 | 1.184 | 0.1213 | Yes |
| 4 | AXL |  |  | 129 | 0.997 | 0.1363 | Yes |
| 5 | MET |  |  | 183 | 0.897 | 0.1510 | Yes |
| 6 | PLAUR |  |  | 185 | 0.893 | 0.1785 | Yes |
| 7 | INHBA |  |  | 196 | 0.876 | 0.2031 | Yes |
| 8 | CD70 |  |  | 253 | 0.819 | 0.2147 | Yes |
| 9 | SERPINE1 |  |  | 299 | 0.756 | 0.2270 | Yes |
| 10 | KLF6 |  |  | 357 | 0.708 | 0.2348 | Yes |
| 11 | LPAR1 |  |  | 421 | 0.668 | 0.2400 | Yes |
| 12 | IL18 |  |  | 427 | 0.666 | 0.2594 | Yes |
| 13 | SLC7A1 |  |  | 448 | 0.651 | 0.2746 | Yes |
| 14 | SPHK1 |  |  | 551 | 0.603 | 0.2681 | No |
| 15 | MYC |  |  | 790 | 0.502 | 0.2249 | No |
| 16 | ADORA2B |  |  | 841 | 0.486 | 0.2276 | No |
| 17 | NFKB1 |  |  | 874 | 0.478 | 0.2345 | No |
| 18 | SGMS2 |  |  | 893 | 0.473 | 0.2447 | No |
| 19 | HBEGF |  |  | 972 | 0.451 | 0.2394 | No |
| 20 | ADRM1 |  |  | 1034 | 0.432 | 0.2377 | No |
| 21 | CDKN1A |  |  | 1088 | 0.419 | 0.2376 | No |
| 22 | IRF1 |  |  | 1173 | 0.403 | 0.2293 | No |
| 23 | PVR |  |  | 1291 | 0.380 | 0.2122 | No |
| 24 | ITGB3 |  |  | 1325 | 0.374 | 0.2157 | No |
| 25 | PCDH7 |  |  | 1329 | 0.373 | 0.2265 | No |
| 26 | IL7R |  |  | 1360 | 0.366 | 0.2304 | No |
| 27 | P2RX7 |  |  | 1383 | 0.360 | 0.2361 | No |
| 28 | RHOG |  |  | 1448 | 0.347 | 0.2311 | No |
| 29 | CD55 |  |  | 1551 | 0.331 | 0.2161 | No |
| 30 | HIF1A |  |  | 1667 | 0.315 | 0.1975 | No |
| 31 | GNAI3 |  |  | 1811 | 0.298 | 0.1714 | No |
| 32 | SLC7A2 |  |  | 1880 | 0.288 | 0.1635 | No |
| 33 | RIPK2 |  |  | 1892 | 0.286 | 0.1697 | No |
| 34 | TPBG |  |  | 1914 | 0.284 | 0.1733 | No |
| 35 | P2RX4 |  |  | 2101 | 0.262 | 0.1355 | No |
| 36 | LDLR |  |  | 2128 | 0.260 | 0.1371 | No |
| 37 | EIF2AK2 |  |  | 2148 | 0.257 | 0.1404 | No |
| 38 | PTPRE |  |  | 2265 | -0.255 | 0.1196 | No |
| 39 | MMP14 |  |  | 2295 | -0.258 | 0.1205 | No |
| 40 | ABI1 |  |  | 2598 | -0.301 | 0.0552 | No |
| 41 | PDE4B |  |  | 2788 | -0.333 | 0.0188 | No |
| 42 | CALCRL |  |  | 3508 | -0.546 | -0.1418 | No |
| 43 | HAS2 |  |  | 3724 | -0.702 | -0.1732 | No |
| 44 | SRI |  |  | 3885 | -0.909 | -0.1845 | No |
| 45 | BTG2 |  |  | 3917 | -0.999 | -0.1612 | No |
| 46 | ITGB8 |  |  | 3962 | -1.093 | -0.1382 | No |
| 47 | CD82 |  |  | 3973 | -1.119 | -0.1060 | No |
| 48 | RGS16 |  |  | 3996 | -1.227 | -0.0734 | No |
| 49 | SLC1A2 |  |  | 4040 | -1.555 | -0.0359 | No |
| 50 | PDPN |  |  | 4046 | -1.612 | 0.0128 | No |
Table: GSEA details [plain text format]

  

Fig 2: HALLMARK\_INFLAMMATORY\_RESPONSE: Random ES distribution      
 Gene set null distribution of ES for **HALLMARK\_INFLAMMATORY\_RESPONSE**
